# Evaluation of Indium Tin Oxide for Gas Sensing Applications: Adsorption/Desorption and Electrical Conductivity Studies on Powders and Thick Films

**DOI:** 10.3390/s21020497

**Published:** 2021-01-12

**Authors:** Stefan Dietrich, Mihails Kusnezoff, Uwe Petasch, Alexander Michaelis

**Affiliations:** 1 Fraunhofer Institute for Ceramic Technologies and Systems IKTS, Winterbergstr. 28, 01277 Dresden, Germany; mihails.kusnezoff@ikts.fraunhofer.de (M.K.); uwe.petasch@ikts.fraunhofer.de (U.P.); 2 Institute of Materials Science, Technische Universität Dresden, 01069 Dresden, Germany; alexander.michaelis@ikts.fraunhofer.de

**Keywords:** indium tin oxide, adsorption, semiconductor gas sensor

## Abstract

By combining results of adsorption/desorption measurements on powders and electrical conductivity studies on thick and thin films, the interaction of indium tin oxide with various ambient gas species and carbon monoxide as potential target gas was studied between room temperature and 700 °C. The results show that the indium tin oxide surfaces exhibit a significant coverage of water-related and carbonaceous adsorbates even at temperatures as high as 600 °C. Specifically carbonaceous species, which are also produced under carbon monoxide exposure, show a detrimental effect on oxygen adsorption and may impair the film’s sensitivity to a variety of target gases if the material is used in gas sensing applications. Consequently, the operating temperature of an ITO based chemoresistive carbon monoxide sensor should be selected within a range where the decomposition and desorption of these species proceeds rapidly, while the surface oxygen coverage is still high enough to provide ample species for target gas interaction.

## 1. Introduction

Indium tin oxide (ITO) is a degenerate n-type semiconductor composed of indium(III) oxide (In_2_O_3_) and tin(IV) oxide (SnO_2_) with a typical mass ratio of 90:10. ITO is stable up to 1600 °C in oxygen but decomposes at temperatures above 1100 °C in nitrogen [1]. Due to its large band gap of approximately 4 eV, thin ITO films exhibit a transmission of up to 90% in the visible range of the optical spectrum. At the same time, its high intrinsic electrical conductivity of up to 1 × 10^4^ S/cm makes the material one of the most important transparent conducting oxides (TCO). As such, it has found extensive application in the fabrication of transparent electrodes for liquid crystal displays (LCD) and touch screens, in organic light emitting diodes (OLED) and as antistatic coating for polymer films. During the past decades, ITO has also seen considerable research as electrode material in gas sensing applications. It has been investigated for the detection of a variety of gases like CO, CO_2_, H_2_, CH_4_, NH_3_, NO, NO_2_ and several more [2,3,4,5,6,7,8,9,10,11,12,13,14,15,16,17,18,19]. While most of these studies focused on ITO thin films, little information is available regarding its use in porous thick films, particularly at temperatures higher than 500 °C [5,6,10,20].

**Figure 1:**
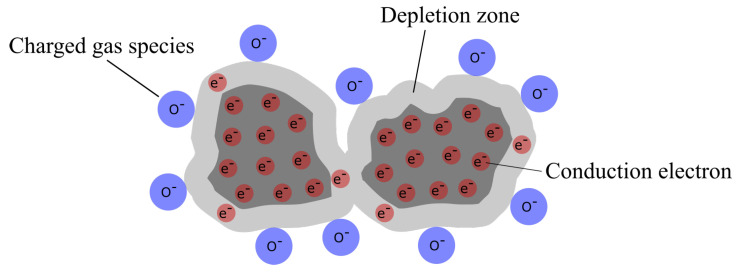
Semiconducting metal oxide particles with charged adsorbates, creating a zone of charge carrier depletion.

In gas sensing applications, the fundamental interaction between gas species and solid surfaces is adsorption. One usually distinguishes between two types of adsorption: physisorption and chemisorption. While physisorbed gas molecules adhere to surfaces only weakly via Van-der-Waals forces, the linking created in chemisorption is similar to chemical bonds. Chemisorbed gas molecules can become electrically charged by the exchange of electrons with the solid surface (often referred to as *ionosorption*) and may also dissociate into charged or uncharged atomic species (*dissociative chemisorption*). If solid surfaces are exposed to oxygen, the gas is usually readily adsorbed and various uncharged (O_2_, O) and charged ($O_{2}^{-}$, O^−^, O^2-^) species are formed depending on surface type, temperature, partial and absolute pressures and further parameters. In the case of n-type semiconducting metal oxides like ITO and its constituent oxides In_2_O_3_ and SnO_2_, surface oxygen species typically reported for temperatures below approximately 150 °C are both physisorbed and chemisorbed molecular oxygen as well as superoxide ions ($O_{2}^{-}$). In the temperature range 200–500 °C, the singly negative charged O^−^ is reported to be the dominant species, while for higher temperatures, the existence of O^2−^ species has been described [21,22,23,24,25,26]. Creating anionic oxygen adsorbates on semiconducting metal oxide surfaces involves the donation of electrons from the conduction band of the material (Figure 1).

Depending on adsorbate coverage, this results in a reduced density of mobile charge carriers in the surface layer of the oxide. The formation of this so-called depletion region has a direct influence on electrical film conductivity and is fundamental for the function of chemoresistive semiconductor gas sensors [27,28,29]. It is interesting to note that as of the time of writing of the present article, direct spectroscopic evidence is sparse or missing for most of the aforementioned charged species. Results of XPS studies on SnO_2_ published in 2018 by Vorokhta, however, appear to prove the existence of O^−^ species [30,31]. At temperatures above approximately 550 °C, oxygen exchange with the crystal lattice may occur [21,30]. If no interaction with other gas species is taking place, desorption of oxygen is temperature-dependent and proceeds either directly via O_2_ molecules or by surface diffusion and recombination of atomic or ionic species. Incident reducing gases like carbon monoxide (CO) primarily interact with adsorbed oxygen. Due to its high reactivity and dominance on the surface, O^−^ is reported to play a major role with three main reactions [30,32]:

(1) $$CO+O_{ads}^{-}\to {CO}_{2,gas}+e^{-}\;({\mathrm{CO}}_{2}\;\mathrm{formation})$$

(2) $$CO+O_{ads}^{-}\to CO_{2}^{-}\;(\mathrm{carboxylate}\;\mathrm{formation})$$

(3) $$CO+2O_{ads}^{-}\to CO_{3}^{2-}\;(\mathrm{carbonate}\;\mathrm{formation})$$

Carboxylate and carbonate species may decompose to CO_2_ and oxygen species via different pathways, during each of which electrons are released back into the oxide surface [32]. In addition, a direct interaction of CO with lattice oxygen has also been described [30]:

(4) $$CO+O_{O}^{\times }⇌{CO}_{2,gas}+V_{O}^{\times }$$

and

(5) $$V_{O}^{\times }⇌V_{O}^{•}+e^{{}^{\prime }}$$

(6) $$V_{O}^{•}⇌V_{O}^{•\;•}+e^{{}^{\prime }}$$

Here, $O_{O}^{\times }$ denotes a neutral lattice oxygen atom, $V_{O}^{\times }$ a neutral oxygen vacancy, $V_{O}^{•}$ and $V_{O}^{••}$ a singly and doubly positive charged oxygen vacancy, respectively, and $2e^{{}^{\prime }}$ an electron. Both the reaction with surface oxygen species as well as a direct interaction with the lattice result in the release of one or more electrons back into the conduction band. This is in agreement with the increase in electrical conductivity, which has been observed under the influence of CO by several authors [3,4,8,33,34].

Despite decades of extensive research, the picture for interactions between gas species and semiconducting oxide surfaces is still incomplete and, with regard to certain mechanisms, even inconclusive. The main reason for this is the broad variety of material compositions, film and surface morphologies and experimental conditions.

In this work, an approach to investigate the interaction between ITO and selected gas species was chosen, which is based on a combination of adsorption/desorption measurements on powders and electrical conductivity studies on thick and thin films. Emphasis was placed on the initial sample state and specific measurement conditions. The studies included the influence of ambient H_2_O, CO_2_ and other carbonaceous species, while O_2_ and CO served as primary target gases in inert atmosphere (N_2_, He).

## 2. Materials and Methods

Commercial ITO powders (Inframat Advanced Materials, Manchester, CT, USA) with an In:Sn ratio specified as 90:10 were acquired. Particle size and shape were evaluated using scanning electron microscopy (Crossbeam NVision40, Carl Zeiss AG, Oberkochen, Germany), while phase composition was determined using X-ray diffraction analysis (XRD) on a D8 Advance (Bruker Corporation, Billerica, MA, USA) with a LaB_6_ standard. Adsorption/desorption measurements were carried out using an AutoChem II 2920 System (Micromeritics Instrument Corp., Norcross, GA, USA) equipped with a ThermoStar GSD 301 T1 quadrupole mass spectrometer (Pfeiffer Vacuum GmbH, Asslar, Germany). Two different measurement techniques were employed: temperature programmed desorption (TPD) and pulse chemisorption measurements. For the experiments, 0.25 g of the sample powder was deposited manually on glass wool inside the sample tube using a stainless steel spatula. Reference measurements were carried out for both techniques, where only glass wool and no sample was used. After each measurement series, both powder and glass wool were discarded and the sample tube cleaned meticulously with different solvents, rinsed in ethanol and distilled water and eventually heated to 120 °C.

TPD measurements were performed in both He and He with 12.5 vol% O_2_ (abbreviated He/O_2_ hereafter) with a constant flow of 20 mL/min at RT–700 °C and a heating rate of 10 K/min. One measurement series consisted of an initial and a repetition run separated only by cooling the sample to room temperature, but without opening the sample compartment or changing the gas atmosphere. For pulse chemisorption measurements, the same base gases and flow rates have been used, with 0.5 ${\mathrm{cm}}^{3}$ test gas pulses consisting of He with 1930 ppm CO dosed into the gas stream at constant temperatures between 350–700 °C (50 K stepping). The above CO concentration is the actual value of the sample gas composition drawn from the cylinder, which was specified with 2000 ppm. Analysis of downstream mass spectrometer signals was limited to gas species showing relevant signals (O_2_, H_2_O, CO and CO_2_).

For electrical studies, two types of samples were used. To allow for determination of ITO electrical conductivity independent of the surrounding gas atmosphere, dense ITO thin films of 60 nm thickness deposited on fused silica substrates with dimensions 100 × 100 × 1 ${\mathrm{mm}}^{3}$ were obtained from an external partner. The films were prepared by magnetron sputtering at 350 °C. In a successive sputtering process, a dense SiO_2_ thin film of 40 nm thickness was deposited on top of the ITO film. The coated substrates were then cut to samples with lateral dimensions of 20 × 7 ${\mathrm{mm}}^{2}$ using a diamond-tipped glass cutter. To study the atmosphere-dependent electrical properties of thick films, pastes were prepared from the ITO powder and screen printed onto 3YSZ substrates (Kerafol GmbH, Eschenbach i. d. Opf., Germany), with four pre-deposited Au thick film pads serving as electrical contacts. The contact pads have lateral dimensions of 5 × 2 ${\mathrm{mm}}^{2}$ and a thickness of approximately 5 µm. The ITO films were fired at 700 °C in air, the resulting porous films have planar dimensions of 7 × 2 ${\mathrm{mm}}^{2}$ and a thickness of approximately 10 µm. For the measurement of the electrical film properties, both thin and thick film samples were placed in a sample holder within a quartz glass tube furnace, electrical connections were made using Au wires and a small amount of Ag and Au conductor paste, respectively. The paste was applied to the cut faces of the thin film samples and the Au contact pads of the thick film samples and then fired at 700 °C in N_2_ for one hour. Gas composition was adjusted using mass flow controllers (Bronkhorst High-Tech B.V., Ruurlo, The Netherlands), while a type 2400 source meter (Keithley Instruments/Tektronix, Inc., Beaverton, OR, USA) was used to record resistance by four-terminal sensing. To obtain precise temperature information during measurement, a type-S thermocouple was placed in close proximity of the sample and read out using an Almemo 2390-3 data logger unit (Ahlborn Mess- und Regelungstechnik, Holzkirchen, Germany).

Thin film reference measurements of electrical conductivity were conducted in a constant gas flow (10 L/h) of N_2_ using the same temperature range and heating rate as in the TPD measurements (RT–700 °C, 10 K/min). With the thick films, the aim was to mimic the conditions of the adsorption/desorption measurements as precisely as possible; therefore, two measuring protocols were used: dynamic measurement with continuous temperature increase and a constant gas flow (10 L/h) of N_2_ or N_2_ with 12.5 vol% O_2_ (abbreviated N_2_/O_2_ hereafter) using the same temperature range and heating rate as with the thin film samples, and static measurements at constant temperature and gas flow (10 L/h), with CO pulses (50–2000 ppm) dosed into the gas stream. Dynamic resistance measurements in each controlled atmosphere were split into two separate runs with the same feed gas composition, temperature ramp and maximum temperature: an initial run after the sample holder with sample had been exposed to ambient lab air for 1 h at room temperature, and a repetition run, which followed the initial run after the sample had cooled to room temperature, but without opening the reactor or changing gas composition. Static measurements with CO pulses at fixed temperature, on the other hand, were performed after heating the sample to 700 °C and successive cooling to room temperature, so the initial conditions of these measurements equal those of the dynamic repetition runs. For both adsorption/desorption and resistance measurements, all gases were supplied from gas cylinders with a 5.0 purity rating, the respective measurements have been carried out at ambient pressure. Before initial measurements, both powders and films (after electrical contacts had been fired) were exposed to ambient lab air for 1 h to establish sample surface conditions as similar as possible.

## 3. Results and Discussion

With the exception of the structural investigations, all adsorption/desorption experiments as well as electrical measurements were repeated 2–3 times with different samples under the same experimental conditions. Unless stated otherwise, the results obtained from these repeated measurements have been consistent.

### 3.1. Structural Powder Properties

**Figure 2:**
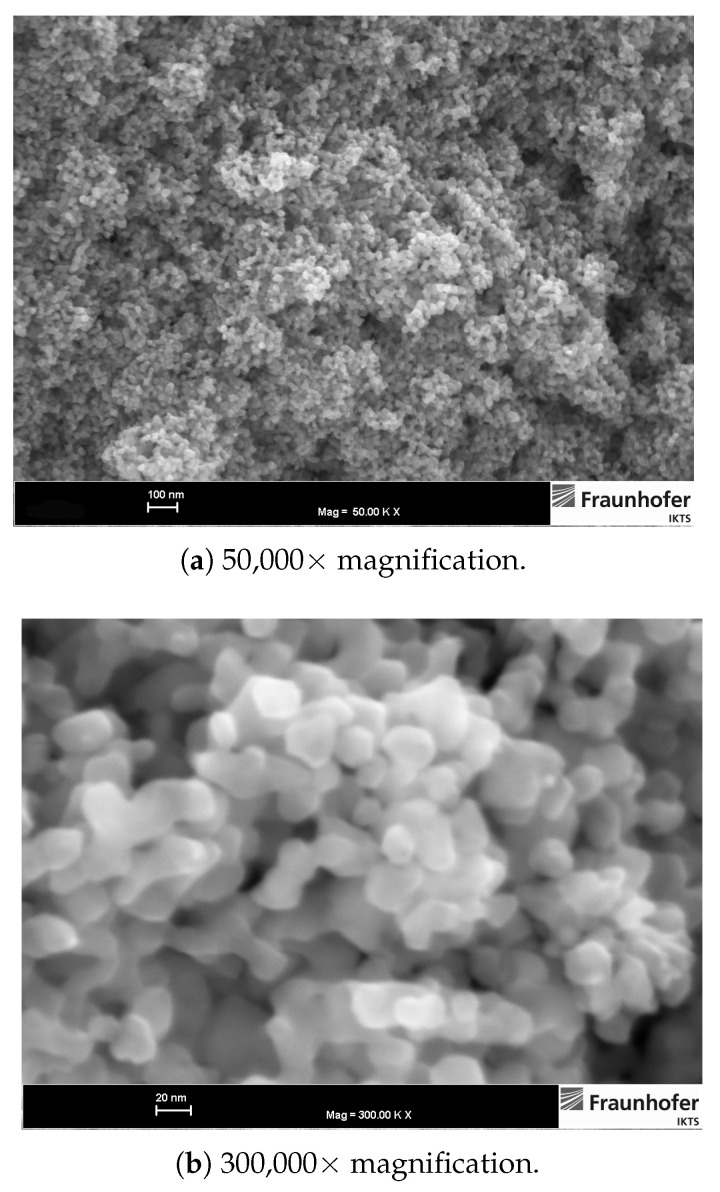
ITO powder, FESEM images of powder surface at two different magnifications.

FESEM images of the ITO powder surface (Figure 2) reveal a narrow particle size distribution. Particles exhibit polyhedral but near spherical shape with a diameter of 10–20 nm. In the manufacturer’s data sheet, the powder’s specific surface area (BET) is denoted as 35.3 $m^{2}/g$, which corresponds to an average particle diameter of approximately 24 nm. This value is in good agreement with the particle diameter estimation based on the FESEM images.

**Figure 3:**
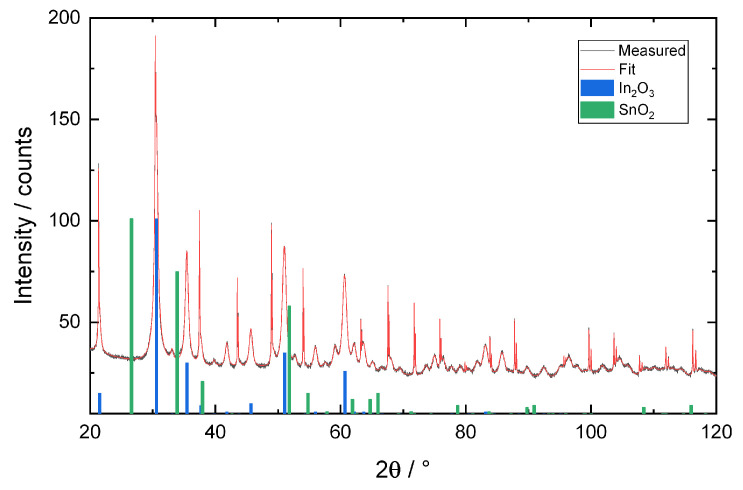
Indium tin oxide (ITO) powder, X-ray diffractogram showing measured (black) and fit data (red). The main peaks for constituent oxides SnO_2_ (green) and In_2_O_3_ (blue) are included for comparison.

Figure 3 shows the XRD diffractogram of the ITO powder. Based on the measured data, a lattice parameter of (10.1303±0.0001) was determined. A comparison with data published by González et al. shows that this lattice parameter corresponds to ITO powder with a Sn content of approximately 9 m% [35]. However, the authors report a high measurement uncertainty for their results. A comparison with diffractograms of the constituent oxides shows an agreement with In_2_O_3_, but not with SnO_2_. This indicates that SnO_2_ is not present as a discrete phase but as a constituent of the In_2_O_3_-SnO_2_ solid oxide solution.

### 3.2. Structural Film Properties

**Figure 4:**
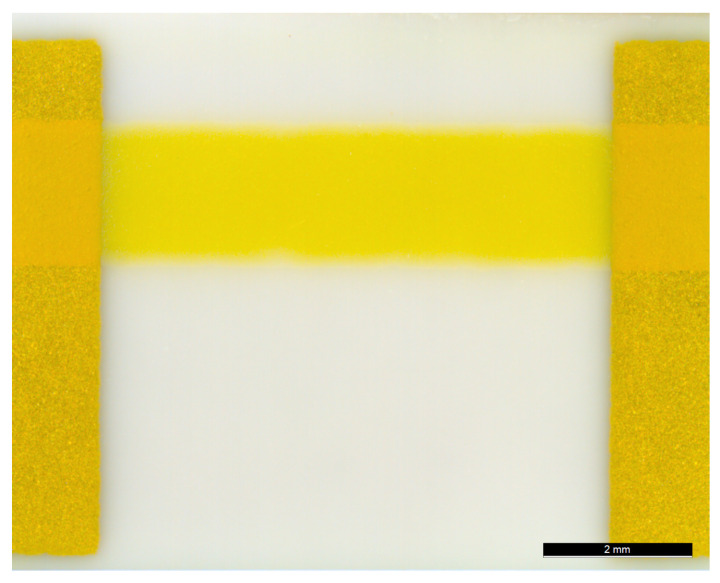
ITO thick film resistance sample, light-optical microscope image at 12-fold magnification. The image shows a part of the film between two of the Au contact pads, deposited on a 3YSZ substrate.

**Figure 5:**
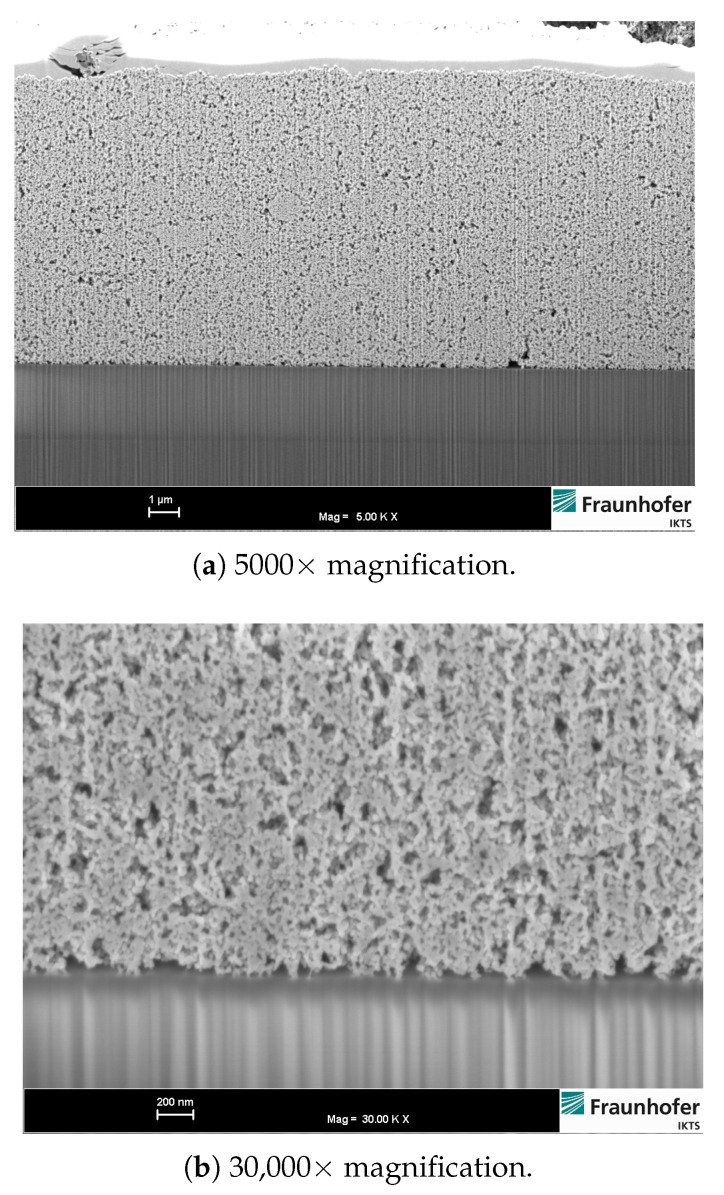
ITO thick film, FESEM images of cross sectional area prepared by focused ion beam (FIB) preparation.

An example of the ITO thick films is illustrated in Figure 4. The figure shows a light-optical microscope image of a part of an ITO thick film deposited on a 3YSZ substrate. Au contact pads are visible on the left and right edge of the frame. In Figure 5, FESEM images of a cross sectional area of an ITO thick film fired at 700 °C are shown. To obtain sufficient cross section quality, samples have been prepared using focused ion beam ablation. The film exhibits a homogeneous structure with open pores and no visible agglomerates. ITO particles at the film-substrate interface show a sufficient number of bonds to the substrate. When comparing with the FESEM images of the ITO powder it becomes obvious that ITO particles in the fired thick film have developed sinter necks and connected conductivity paths.

### 3.3. Interaction with Ambient Species

#### 3.3.1. Reference TPD Measurements

**Figure 6:**
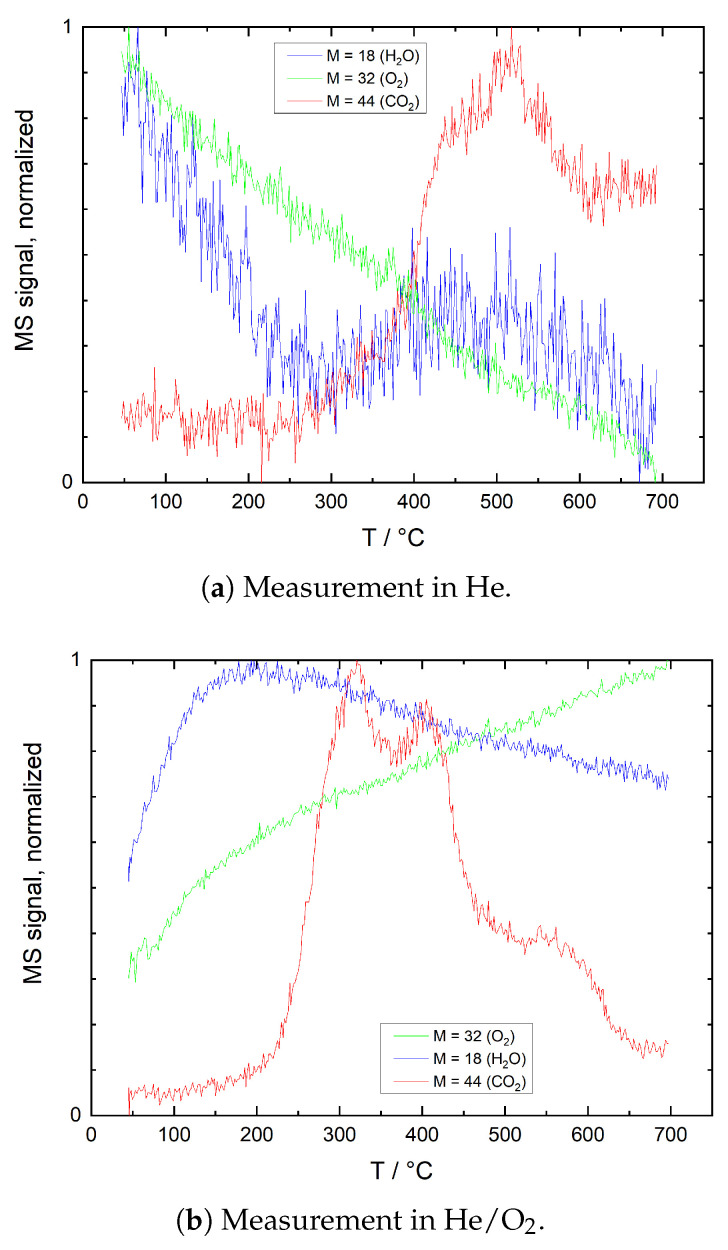
TPD plots, reference measurements without sample in (**a**) He and (**b**) He + 12.5 vol% O_2_ (He/O_2_). Mass spectrometer signals for mass numbers 32 (O_2_), 18 (H_2_O) and 44 (CO_2_) have been normalized individually for better comparability.

Figure 6 shows TPD reference results obtained without sample powder in He and He/O_2_. Neither of the plots shows relevant O_2_ desorption peaks. This is particularly interesting as both sample tube and glass wool had been exposed to ambient air prior to the measurement. Furthermore, even the constant He flow contains approximately 10–20 ppm of oxygen due to intrinsic impurities and small gas line leakages. Although oxygen adsorption on SiO_2_ surfaces has been reported by several authors [36,37,38], it can be concluded that under the present measurement conditions, it either plays no significant role, or the desorption of the resulting surface species is strongly inhibited.

For H_2_O, only a weak peak at 300–500 °C is detected in He, which may be related to the recombination and desorption of OH^−^ groups [37]. However, the peak amplitude is only in the order of magnitude of signal noise. In He/O_2_, the onset of desorption at room temperature and the maximum at 190 °C suggest that a significant amount of the detected water is related to previously physisorbed H_2_O molecules [37]. Water, though not a component of the gas mixtures used, is assumed to adsorb at the walls of the glass tube and the glass wool during exposure to ambient air prior to the measurement.

CO_2_ desorption peaks, on the other hand, may be attributed to a variety of adsorbates. Besides molecularly adsorbed CO_2_, these may be oxidation or decomposition products of contaminants from ambient air or impurities of the test gas like hydrocarbons, carboxylates and carbonates or even graphitic carbon. For most of these species, oxidation to CO_2_ requires a temperature of at least 150 °C even with an effective catalyst [39,40,41]. Considering a desorption onset at temperatures as high as 200 °C (He) and 300 °C (He/O_2_), desorption of molecularly adsorbed CO_2_ appears unlikely. The shift of desorption maxima to lower temperatures in an oxygen-rich environment indicates that these peaks can indeed be attributed to oxidation of carbonaceous contaminants.

#### 3.3.2. TPD Measurements on ITO Powder

**Figure 7:**
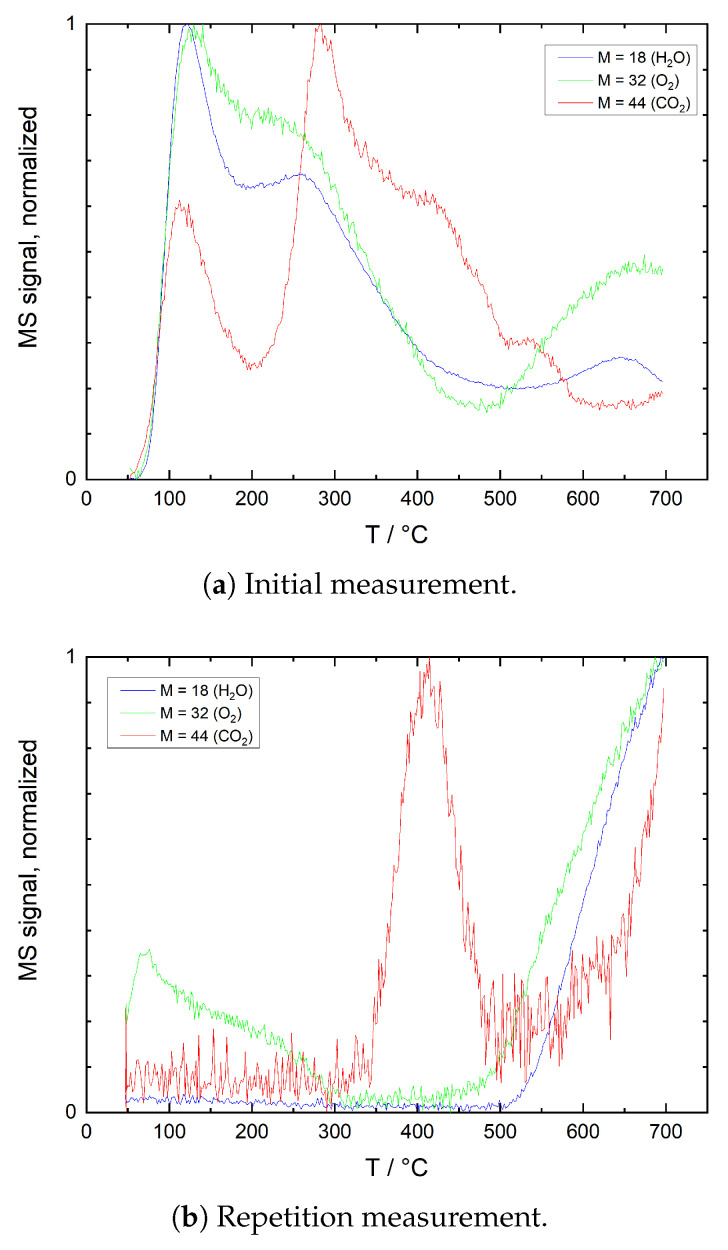
TPD plots of ITO powder in He, showing normalized mass spectrometer signals for mass numbers 32 (O_2_), 18 (H_2_O) and 44 (CO_2_). (**a**) Initial measurement after 1 h of powder exposure to ambient air at room temperature, (**b**) repetition measurement after cooling to room temperature following the initial measurement, without changing gas composition.

**Figure 8:**
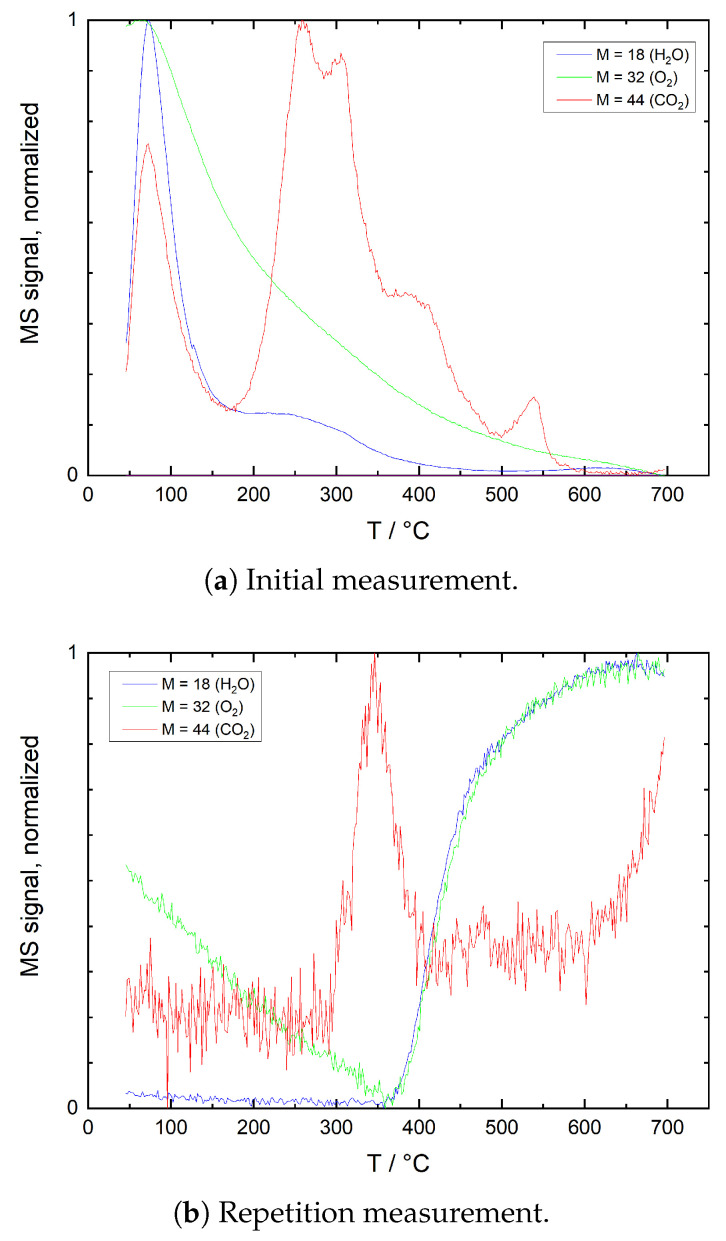
TPD plots of ITO powder in He + 12.5 vol% O_2_ (He/O_2_), showing normalized mass spectrometer signals for mass numbers 32 (O_2_), 18 (H_2_O) and 44 (CO_2_). (**a**) Initial measurement after 1 h of powder exposure to ambient air at room temperature, (**b**) repetition measurement after cooling to room temperature following the initial measurement, without changing gas composition.

Figure 7 shows results of TPD measurements on ITO powders in He. Multiple desorption peaks are visible for O_2_, H_2_O and CO_2_ in the initial measurement, while the second run exhibits a distinct CO_2_ peak and an increase of all signals with an onset at around 500 °C. Figure 8 shows TPD measurement results of ITO powder in He/O_2_. Again, in the first run, several desorption maxima are visible for H_2_O and CO_2_, while the O_2_ signal shows a continuous decline. In the second run, a discrete CO_2_ peak appears at 350 °C and both H_2_O and O_2_ signals start increasing at around 370 °C.

The comparison with reference measurements without sample reveals only little agreement. In He/O_2_ the low-temperature regions of the CO_2_ double peaks at 200–300 °C appear similar, but signal amplitudes and signal-to-noise ratio differ significantly. TPD measurements on ITO powder are therefore only little affected by background effects of the test bench. However, the similar temperatures at which some of the CO_2_ peaks are detected may be indicative that for both test bench and sample powder similar species and contaminants contribute to the signal.

#### 3.3.3. R(T) Measurements on ITO Films

**Figure 9:**
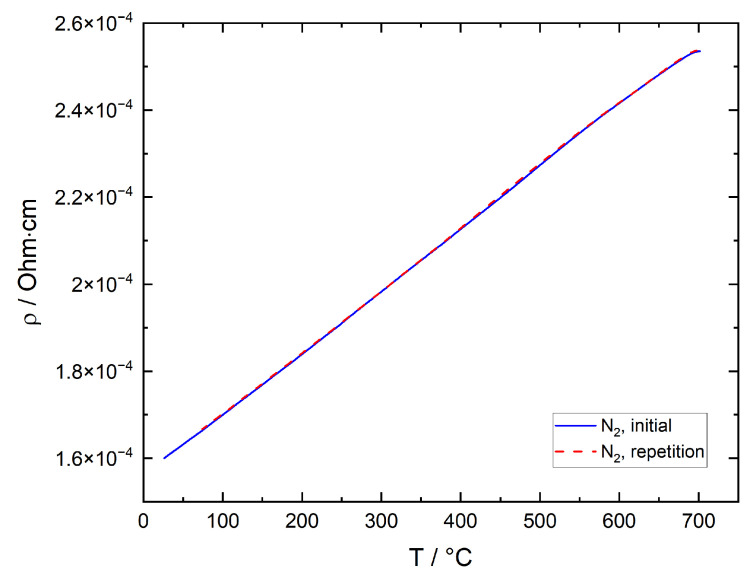
Electrical resistivity of ITO thin film as a function of temperature measured in N_2_.

The typical resistance of the thin films during measurements is approximately 75–125 Ohms. In Figure 9, results of the R(T) measurements on SiO_2_ covered ITO thin films in N_2_ are illustrated. Resistivity increases almost linearly until the temperature reaches 700 °C. No substantial difference in resistivity can be seen between initial measurement after ambient air exposure and repetition measurement.

**Figure 10:**
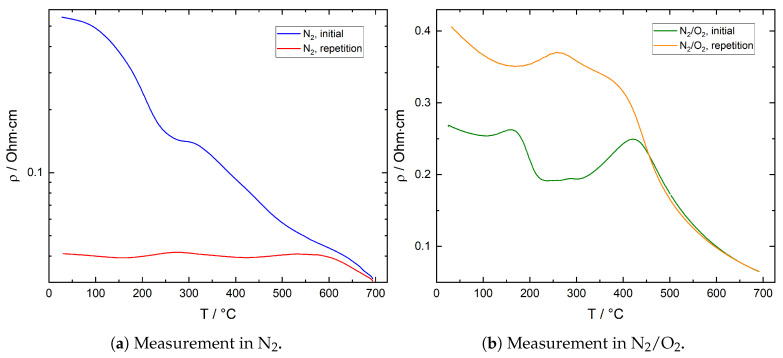
Electrical resistivity of ITO thick film as a function of temperature measured in (**a**) N_2_ and (**b**) N_2_ + 12.5 vol% O_2_ (N_2_/O_2_). Initial measurement after 1 h of film exposure to ambient air at room temperature, repetition measurement after cooling to room temperature following the initial measurement, without changing gas composition. In (**a**), a logarithmic resistivity axis scale was used.

The temperature and atmosphere dependent resistance of the ITO thick films is typically in the range of approximately 100–2000 Ohms. Figure 10 shows results of the R(T) measurements on an ITO thick film in N_2_ and N_2_/O_2_ for a typical sample. To a good approximation, the results for three different film samples were identical under the same measurement conditions. The same applies for repeated cycles of initial and repetition measurements over the course of several weeks.

During the initial measurement in inert atmosphere after ambient air exposure, resistance is declining continuously except for a shallow plateau at around 250 °C. In the second measurement, resistance is significantly lower than in the initial measurement and exhibits various local maxima and minima before a significant decrease is observed at temperatures above 600 °C. On the contrary, in oxygen-rich atmosphere, resistance at temperatures below 450 °C is considerably lower in the initial measurement than in the second measurement. Here, a significant local resistance minimum is observed between 200 and 350 °C, followed by a resistance maximum and a sharp drop-off at above 450 °C. The second measurement reveals a local resistance minimum at 170 °C, followed by a shallow maximum and a resistance decrease beginning at 250 °C. Both resistance plots are nearly identical between 450 and 700 °C. As expected, the film resistivity measured during the repetition measurement in N_2_ is significantly lower than during measurement in N_2_/O_2_ over the entire temperature range due to the lower oxygen partial pressure in the inert atmosphere and subsequent lower surface oxygen coverage. It is worth pointing out that even the lowest thick film resistivity of approximately 4 × 10^−2^ Ohm·cm, which is found at room temperature after previously heating the sample to 700 °C in N_2_, is roughly 250 times higher than the thin film’s resistivity at the same temperature. The main reason for this discrepancy lies in the porous structure of the thick film in contrast to the dense thin film. In the thick film, intrinsic film resistance is controlled by direct particle–particle contacts of a relatively small cross sectional area and thus a high resistivity.

#### 3.3.4. Combining TPD and R(T) Results: The General Picture

##### Resistance Measurement on ITO thin Films in N_2_

The measured resistivity range agrees well with data found in the literature, where electrical conductivity is documented with values of up to 10^4^ S /cm [42,43,44]. With a In_2_O_3_:SnO_2_ mass ratio of 90:10, the ITO used in the present studies is a heavily degenerate semiconductor. Materials of this class exhibit electrical conductivity behavior, which resembles that of metallic conductors rather than that of semiconductors, which is why resistivity increases with increasing temperature. In addition, the almost linear dependence of resistivity on temperature over the whole measuring range and the nearly equivalent plots for initial and repetition measurement contrast the results other authors have published for sputtered ITO thin films [9] and show that the influence of gas species from ambient air exposure is negligible due to the protective SiO_2_ coating.

##### Initial Measurement in N_2_

After exposure to ambient air, the initial drop in resistance between room temperature and 250 °C coincides with significant desorption of O_2_, H_2_O and CO_2_. In this temperature range, the desorption of charged oxygen species typically found on ITO and its constituent oxides leads to a resistance decrease. Desorption of chemisorbed CO_2_ was found to cause the resistance to increase in additional measurements and by other authors [6,20]. Desorption of chemisorbed water is associated with an increase in resistance as well [5]; however, at temperatures below 150 °C, water prevails as molecularly adsorbed species [33]. The observed drop in resistance is therefore dominated by desorption of chemisorbed oxygen, while the detected H_2_O and CO_2_ originates mainly from physisorbed species. At 250–350 °C, the CO_2_ desorption peak observed in inert atmosphere agrees well with the shallow plateau in resistance in the same temperature range. Both the oxidation of carbonaceous contaminants with adsorbed oxygen as well as the decomposition of carboxylates and carbonates are expected to cause a decrease in resistance, while the plateau in the data indicates an approximately constant resistance.

**Figure 11:**
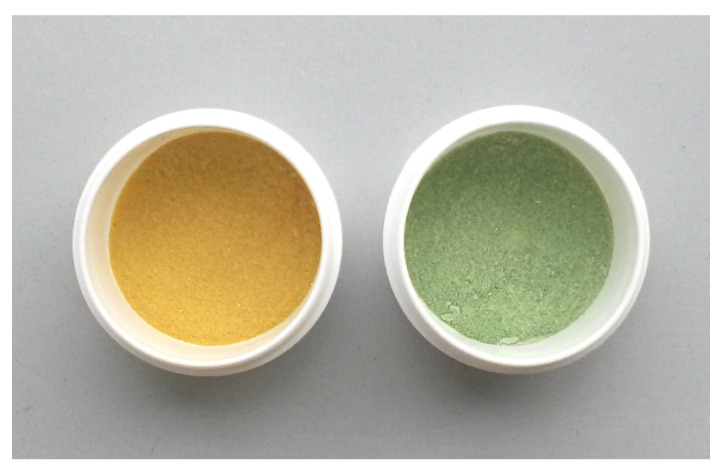
ITO powder sample, before (**left**) and after (**right**) TPD in He, showing a discoloration from yellow to greenish-gray.

It is assumed that in this temperature range chemisorbed CO_2_ desorbs, which partially counteracts the resistance decrease caused by oxygen desorption. At 300 °C and above, resistance decreases again, which can be partially attributed to further oxygen desorption. At higher temperatures, an additional contribution of the hydroxyl group recombination mechanism described by Egashira et al. is assumed. According to the authors, OH^−^ recombine to form H_2_O and O_2_, which desorb releasing two electrons. This hypothesis is in good agreement with the coincidence of the H_2_O and O_2_ peaks between 500 and 650 °C [45]. Furthermore, release of lattice oxygen may also result in a resistance decrease. Although no direct evidence for the removal of lattice oxygen could be established, the discoloration of the ITO powder during TPD in inert atmosphere shown in Figure 11 is an indication of an at least surficial reduction: in literature, a grayish to brown-blackish discoloration has been reported for ITO powder treated under low oxygen or reducing atmospheres [46,47,48]. On the other hand, Bronin et al. could show that the exchange of oxygen between surface and bulk of In_2_O_3_ is negligible even at temperatures as high as 900 °C [49]. It is therefore assumed that under the present measurement conditions, the removal of lattice oxygen is constricted to the surface region of the material. The resistance decrease by both hydroxyl group recombination and the release of lattice oxygen dominates the electrical conductivity with rising temperature. When comparing these results with the reference resistance measurement on SiO_2_ coated ITO thin films shown in Figure 9, it becomes obvious that the total electrical conductivity of the thick film is controlled by the electrical resistance of the contacts between individual particles (grain boundaries) and the interaction with gas species over the whole temperature range between room temperature and 700 °C.

##### Repetition Measurement in N_2_

In the second measurement, oxygen desorption is detected between room temperature and 300 °C. Both amplitude and signal-to-noise ratio are low, at the same time no clear correlation with resistance in this temperature range is observed. The desorbing oxygen is therefore attributed to species adsorbed from oxygen traces in the base gas during cool-down after the initial measurement and the early heating phase of the repetition measurement. The prominent CO_2_ peak visible in the TPD plots coincides with the weak local resistance minimum visible at 420 °C. However, the TPD peak shows a low amplitude and signal-to-noise ratio. As chemisorbed CO_2_ can be ruled out at this temperature and due to the sign of resistance change, the effects are an indication of oxidation of carbonaceous contaminants and/or decomposition of carbonates, which have not been fully eliminated during the initial measurement or re-deposited from the carrier gas during cooling of the sample. The resistance decrease observed at 600 °C and above is again attributed to recombination of hydroxyl groups, which agrees well with the TPD signals for O_2_ and H_2_O in this temperature region. Obviously, some of the hydroxyl groups are bound strongly enough to only desorb after repeated heating to temperatures above 600 °C. Again, this effect and the possible release of lattice oxygen dominate over the decrease in intrinsic electrical conductivity with increasing temperature.

##### Initial Measurement in N_2_/O_2_

After ambient air exposure, a decrease in resistance is observed in N_2_/O_2_ at temperatures of up to 110 °C. TPD diagrams (Figure 8a) show significant H_2_O and CO_2_ desorption in this temperature region, while no significant oxygen desorption is detected. As the desorption of chemisorbed H_2_O and CO_2_ would cause a resistance increase, it is assumed that these species are mainly present as physisorbed species in this temperature region and that the resistance decrease is attributed to the increase in intrinsic electrical conductivity of the ITO film. The distinct resistance minimum at 200–350 °C coincides with a CO_2_ desorption peak; however, desorption of chemisorbed CO_2_ would again result in a resistance change with opposite sign. It is therefore assumed that in this temperature range, oxidation of carbonaceous contaminants proceeds, consuming chemisorbed oxygen and thus releasing electrons back into the semiconductor surface. The resistance drop observed at temperatures above 450 °C indicates at desorption of remaining oxygen, and although resistance appears to trail off exponentially, within the temperature range studied, the decrease in intrinsic electrical film conductivity is concealed by the influence of adsorbates.

##### Repetition Measurement in N_2_/O_2_

During the measurements in N_2_/O_2_, oxygen partial pressure is considerably lower than during ambient air exposure prior to the initial measurement (12.5 vs. 21 vol%). Therefore, surface oxygen coverage and thus resistance is assumed to be higher before than after the initial measurement, as oxygen is expected to desorb from the surface due to the lower oxygen concentration of 12.5 vol% during measurement. However, the results show exactly the opposite resistance behavior, with resistance in the second measurement being significantly higher at temperatures below 450 °C than in the initial measurement. This is indicative of a competition between O_2_ and ambient H_2_O and CO_2_ as well as other carbonaceous species for adsorption sites on the ITO surface, which are adsorbed during ambient air exposure prior to the initial measurement. The result is a lower oxygen coverage and as a consequence a lower resistance unless these species desorb. While no distinct correlation between resistance and TPD measurement results (Figure 8b) is obvious at temperatures below 400 °C, the resistance decrease beginning at 400 °C is likely to be caused by recombination of hydroxyl groups, which again is in good agreement with TPD results. With further temperature increase, resistance is dominated by the desorption of remaining oxygen. Again, electrical film conductivity is controlled by adsorbates up to the highest temperature studied, and the intrinsic electrical conductivity behavior has no significant impact on measurement results.

#### 3.3.5. Summary and Comparison with Literature

**Figure 12:**
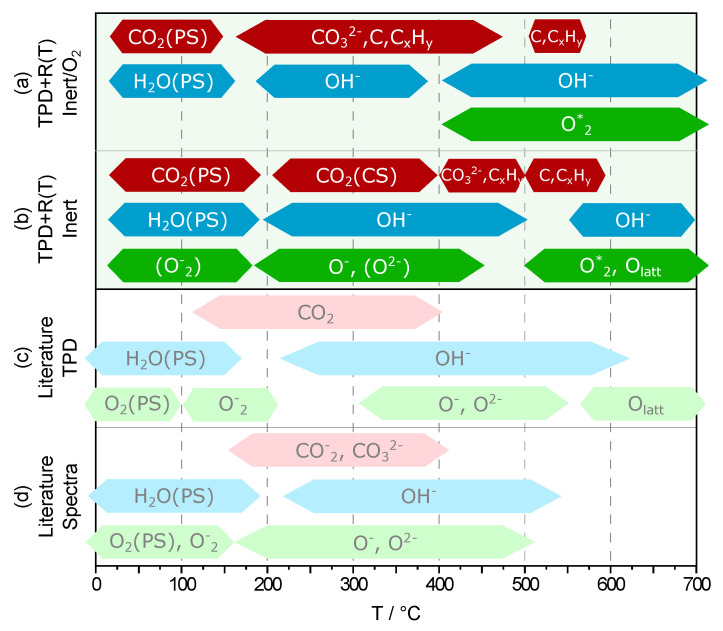
Overview of ITO surface species and corresponding temperature ranges identified by combining results of TPD and electrical resistance measurements for (a) inert and (b) oxygen rich atmosphere, compared to literature data for ITO, SnO_2_ and In_2_O_3_ obtained from (c) TPD and (d) spectroscopic studies. PS and CS refer to physisorbed and chemisorbed species, respectively, C denotes graphitic carbon, C_x_H_y_ hydrocarbons and $O_{2}^{*}$ oxygen formed by the recombination of hydroxyl groups. A species in parentheses indicates that the measurement results do not provide sufficient information to identify the particular species.

A direct comparison of the results with data published in literature is complicated by several factors: first, TPD results so far have only been published for SnO_2_ and In_2_O_3_, but not for ITO [23,50,51]. Second, in most articles, precise information regarding sample history and measurement conditions, for example whether dry synthetic or ambient air was used, is incomplete or missing. Third, temperature ranges given for the presence of a particular species on the respective oxide surfaces vary by up to 100 °C, which is partially due to the different analytical methods used. Therefore, the literature data illustrated in the overview given by Figure 12 correspond to temperature ranges for which the data found for ITO, SnO_2_ and In_2_O_3_ show the best agreement [5,21,22,23,24,25,26,29,32,33,45,50,51,52,53,54,55,56,57,58,59,60,61]. The most important findings for the relevant species are summarized as follows:

- **Oxygen**
  The surface spectra published in literature reveal oxygen species only at temperatures of up to 500 °C. Consequently, oxygen detected in TPD studies at higher temperatures is attributed mainly to the removal of lattice oxygen. While it has been shown that lattice oxygen does contribute to the measured desorption peaks, this explanation falls short when the amount of desorbed O_2_ is considered. The H_2_O detected at the same time reveals that a significant portion of the detected oxygen does not originate from discrete oxygen adsorbates, but from the recombination of surface hydroxyl groups.
- **Water**
  While the low-temperature results for H_2_O agree well with literature data, on both powder and films used in the present studies, hydroxyl groups have been found at temperatures of up to 700 °C, which is roughly 100 °C higher than reported in literature. Furthermore, the results show that the hydroxyl group recombination postulated by Egashira et al. [45] has to be taken into account as a significant desorption mechanism at high temperature.
- **Carbonaceous species**
  CO_2_ physisorbed from ambient air is a relevant surface species between room temperature and approximately 200 °C, a finding which contrasts the low temperature results published by other authors. In addition, the presence of hydrocarbons and graphitic carbon has not been reported for temperatures above 400 °C so far. However, the results show that these species may contribute to the surface coverage at temperatures as high as 600 °C. As with hydroxyl groups, this finding underlines the importance of proper sample preparation after exposure to non-inert atmospheres.

### 3.4. Interaction with CO Pulses

#### 3.4.1. Reference Measurements without Sample

**Figure 13:**
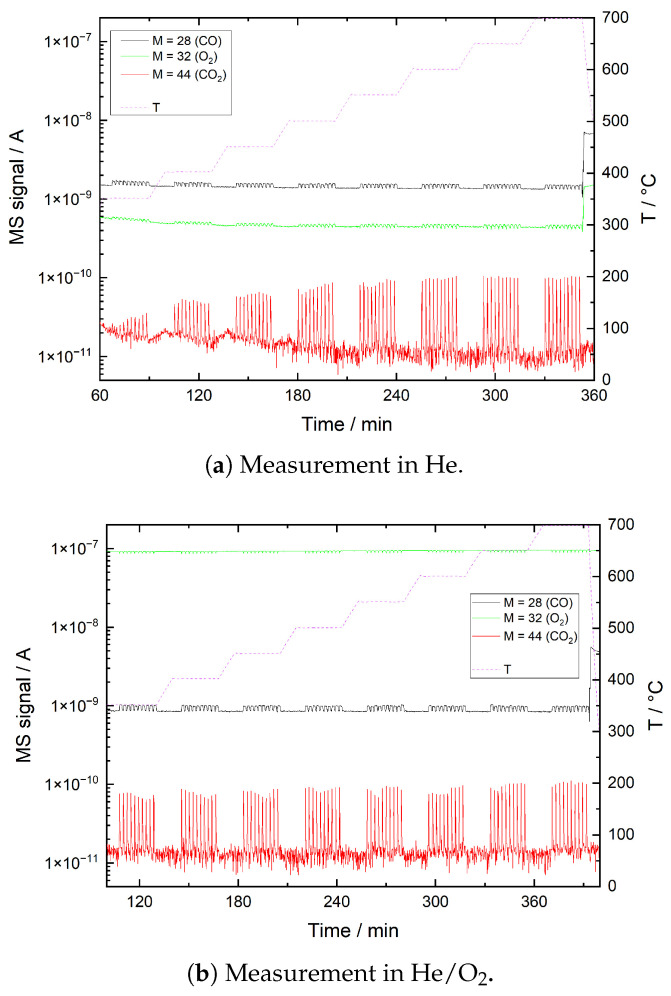
CO pulse measurement, reference measurement without sample recorded in (**a**) He and (**b**) He + 12.5 vol% O_2_ (He/O_2_) at 8 different temperature levels. The plots show mass spectrometer signals for mass numbers 28 (CO), 32 (O_2_) and 44 (CO_2_).

In Figure 13, results of reference CO pulse measurements in He and He/O_2_ are plotted, respectively. The plots show CO peaks, which means that the CO of the pulses is neither adsorbed nor oxidized entirely if no sample is present. As it is not plausible that all of the detected CO_2_ originates from adsorbed CO_2_ or oxidation of contaminants, significant oxidation of incident CO can be concluded, especially at temperatures above 450 °C. In He the oxygen may originate from both adsorption during ambient air exposure and from minor gas line leakages as well as impurities in the test gas. This finding appears contradictory to the results of the TPD measurements, where no significant oxygen desorption from the glass components of the test bench was detected. It is therefore assumed that oxygen does in fact adsorb on the surface of the sample tube and glass wool. However, it appears that while its thermally activated desorption is inhibited, the interaction with CO is not. This is supported by the findings of Berger and Rotzoll, who demonstrated that CO oxidation on glass surfaces is a possible mechanism [62]. Due to the high surface area compared to the tube walls, this process takes place primarily on the surface of the glass wool.

#### 3.4.2. Measurements on ITO Powder

**Figure 14:**
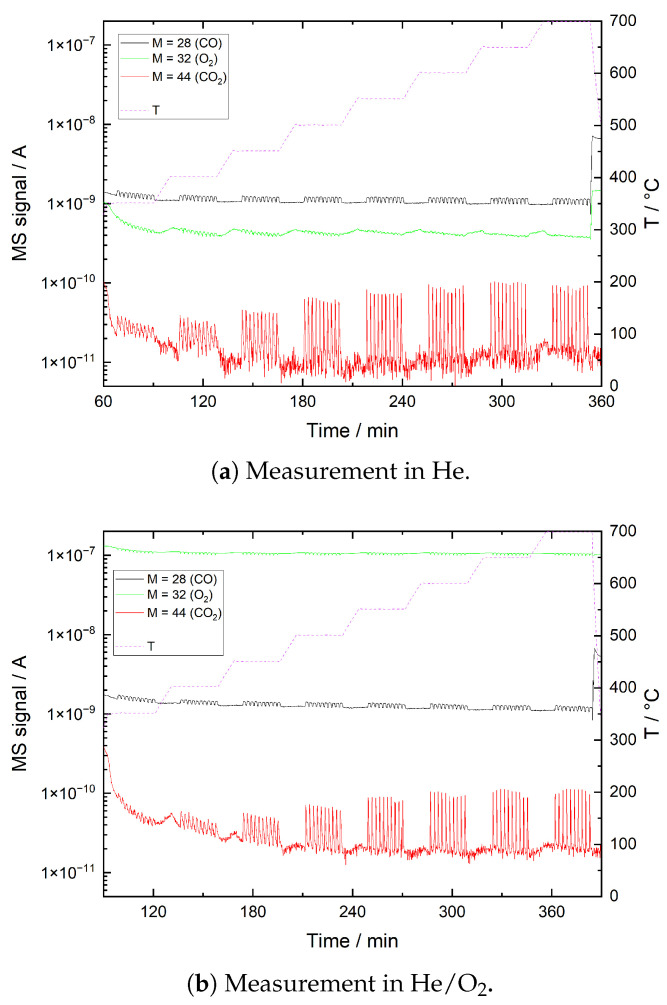
CO pulse measurement, ITO powder, recorded in (**a**) He and (**b**) He + 12.5 vol% O_2_ (He/O_2_) at 8 different temperature levels. The plots show mass spectrometer signals for mass numbers 28 (CO), 32 (O_2_) and 44 (CO_2_).

In Figure 14, the results of the CO pulse measurements in He and He/O_2_ are plotted, respectively. Again, the detected CO peaks show that neither in He nor in He/O_2_ the CO of the pulses is entirely adsorbed or converted. However, in both atmospheres part of the CO reacts to form CO_2_. With the number of CO molecules per pulse estimated to 3 × 10^16^ and assuming a coverage limit of 10^13^ molecules /cm^2^ given for chemisorbed species by Weisz based on the donor/acceptor density in semiconductors [63], the sample surface would already be saturated with 4 × 10^17^ CO molecules. Considering that a significant portion of the surface is already covered with other species, it is reasonable to assume that the actual CO surface coverage is significantly lower than given by this theoretical limit. The sample surface will thus be saturated by less CO than is available per pulse, which manifests in CO slip detected for each pulse.

#### 3.4.3. ITO Film Resistance under CO Influence

**Figure 15:**
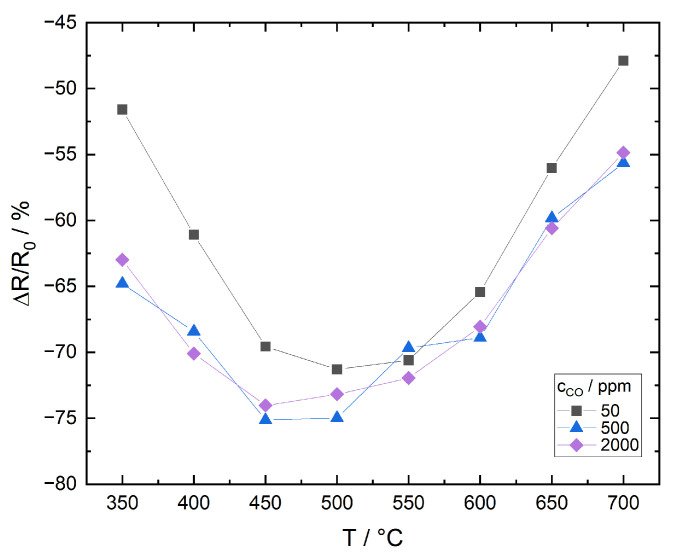
Relative resistance change of ITO thick film as a function of temperature for 3 different CO concentrations, measured in N_2_.

**Figure 16:**
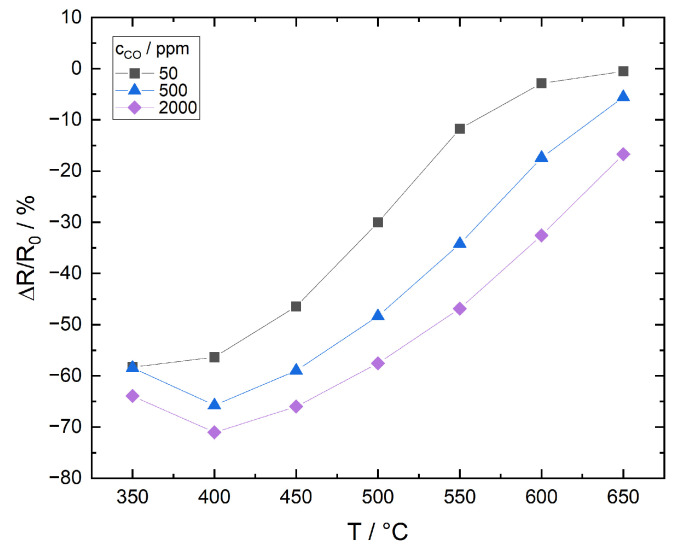
Relative resistance change of ITO thick film as a function of temperature for 3 different CO concentrations, measured in N_2_/O_2_.

Figure 15 shows the relative resistance change as a function of temperature for three CO concentrations in N_2_, Figure 16 for N_2_/O_2_. Here, $R_{0}$ is the temperature dependent initial resistance in the absence of CO, which is in the range of approximately 85–120 Ohms. For the following detailed discussion it is worth pointing out that the ITO mass in the thick films equals only around 0.3% of the powder mass used for adsorption/desorption measurements. According to the previous approximation for maximum surface coverage, the films will theoretically be already saturated after 1 s when 50 ppm CO are supplied in the continuous gas flow.

##### Measurement in N_2_

The weak dependence of resistance change on CO concentration is another indication that the film surface is already saturated with low CO concentrations. Although the concentration of oxygen contamination is only around 20 ppm in the test gas, oxygen may adsorb during the whole measuring cycle due to the continuous gas flow.

The following model for the CO sensitivity of the film is proposed:

- T<450 °C: CO adsorbs at the surface and reacts with oxygen species to form carbonates and carboxylates. Decomposition of these species to CO_2_, which is the step relevant for charge transfer, is inhibited due to insufficient thermal activation. The film’s CO sensitivity is low.
- T=450–500 °C: Carbonates and carboxylates, which are formed by the reaction of CO with adsorbed oxygen, decompose rapidly releasing electrons to the conduction band and forming CO_2_, which desorbs to the gas phase. Film sensitivity exhibits a maximum.
- T>500 °C: Residual oxygen desorbs from the surface so that only a small coverage is still available for interaction with CO. Even at high temperatures, the interaction of CO with lattice oxygen has no significant impact on film resistance. Film sensitivity decreases.

##### Measurement in N_2_/O_2_

The relative change of resistance in N_2_/O_2_ is in the range 0–70%, while it is 50–75% in N_2_. Sensitivity is therefore marginally higher in N_2_, but resolution is significantly lower. The effects can be described with the following model:

- T=400 °C: CO reacts with adsorbed oxygen to carbonates and carboxylates. The sensitivity maximum suggests an increased release of electrons to the conduction band; however, in the CO pulse measurements, little CO_2_ desorption is detected at T ≤ 400 °C. This appears to contradict with the mechanism described in literature, according to which electron release takes place when the carbonates and carboxylates formed by CO interaction with oxygen adsorbates decompose and the resulting CO_2_ desorbs. It is therefore assumed that decomposition and electron release take place, but desorption of the produced CO_2_ is inhibited or retarded.
- T>400 °C: Similar to the measurement in N_2_, oxygen desorbs from the surface. Despite the high availability in the sample gas, only a small oxygen coverage remains for interaction with CO. As in N_2_, the interaction with lattice oxygen plays no significant role. Film sensitivity decreases.

## 4. Conclusions

At low temperatures, the surface of ITO powders and thicks films is not only host to oxygen adsorbates, but exhibits a significant coverage of various species that adsorb during ambient air exposure or from contaminations of the measuring gas. Notable adsorbates besides water and hydroxyl groups are carbon dioxide and other carbonaceous species, some of which are stable at temperatures of up to 700 °C. A comparison with the temperature dependence of resistance obtained from measurements on SiO_2_ covered ITO thin films shows that ITO thick film resistivity is controlled by inter-particle contact resistances and adsorbates over the whole temperature range studied. These species inhibit the adsorption of oxygen and lead to a lower catalytic activity towards the oxidation of CO. In addition, the decomposition of carbonates and carboxylates formed by the reaction of incident CO with adsorbed oxygen is slow and incomplete at temperatures below 400 °C, which furthers restricts the surface available for gas–solid interactions. Accordingly, the sensitivity of film resistance to CO shows a maximum at 400–550 °C, which is the range where decomposition and desorption of carbonaceous species proceeds rapidly enough, but surface oxygen coverage is still sufficiently high. While the ideal operating conditions of chemoresistive sensors based on ITO thick films still depend on several parameters like oxygen and target gas concentrations, the results may provide a guideline for choosing the temperature range best suited for reproducible CO detection.

## Data Availability

The data presented in this study are available on request from the corresponding author.

## Abbreviations

The following abbreviations are used in this manuscript:

ITO	Indium tin oxide
BET	Brunauer–Emmett–Teller
FESEM	Field emission scanning electron microscope
OLED	Organic light emitting diode
TCO	Transparent conducting oxide
TPD	Temperature programmed desorption
XPS	X-ray photoelectron spectroscopy
XRD	X-ray diffraction
